# Computation Offloading Strategy Based on Multi-Agent Reinforcement Learning in Vehicular Edge Computing Networks

**DOI:** 10.3390/s26092652

**Published:** 2026-04-24

**Authors:** Yubao Liu, Quanchao Sun, Zhiyuan Liu

**Affiliations:** College of Computer Science and Technology, Changchun University, Changchun 130022, China; 241503556@mails.ccu.edu.cn (Q.S.); liuzy@ccu.edu.cn (Z.L.)

**Keywords:** vehicular edge computing, multi-agent reinforcement learning, computation offloading, cross-zone communication, Transformer

## Abstract

With the development of intelligent transportation systems, vehicular applications demonstrate diverse characteristics, including computation-intensive processing and stringent latency requirements. Traditional computation offloading strategies struggle to cope with the highly dynamic, multi-node, and multi-task concurrent vehicular network environment and generally overlook the risk of cross-zone communication failures caused by high-speed mobility. To address this issue, this paper designs a computation offloading algorithm based on multi-agent reinforcement learning. This method comprehensively considers four heterogeneous features including queue load, communication links, task attributes, and computing resources, establishes a multi-layer collaborative computing architecture integrating task migration and result return mechanisms, and further constructs an optimization model aimed at minimizing the weighted sum of latency and energy consumption. This model is formalized as a multi-agent Markov decision process, and an improved Multi-Agent Proximal Policy Optimization(MAPPO)-based MATPPO-T algorithm is designed to solve it, achieving one-step joint optimization of task offloading, resource allocation, and task result migration. Experimental results demonstrate that the proposed method reduces the total system cost by approximately 22% on average compared to benchmark algorithms such as MAPPO and PPO, while consistently maintaining the lowest offloading overhead and fastest convergence speed, validating its robustness and scalability in dynamic vehicular edge networks.

## 1. Introduction

In recent years, driven by the dual advancement of artificial intelligence and wireless communication technologies, traditional transportation systems have gradually evolved toward intelligent transportation systems [1]. With the increasing number of connected autonomous vehicles, various computation-intensive and latency-sensitive applications are emerging, such as image-assisted navigation and augmented reality (AR) driving [2]. However, existing resource-constrained vehicles cannot fully satisfy these low/ultra-low latency requirements [3].

Traditional centralized mobile cloud computing (MCC) and distributed fog computing paradigms are constrained by the physical distance of resource deployment and architectural characteristics, commonly suffering from high end-to-end latency and significant device energy consumption, making it difficult for them to meet the high-performance requirements of vehicular networks for low latency, high reliability, and high dynamics [4]. In contrast, mobile edge computing (MEC) addresses this by deploying core resources such as computation and storage to the network edge, providing proximity services to end users and physically shortening data transmission paths, thus becoming a key technical direction to resolve the contradiction between low latency and low energy consumption [5]. By integrating vehicular networks into mobile edge computing (MEC), a new paradigm named vehicular edge computing (VEC) has emerged [6], which extends the MEC architecture to road scenarios. Through edge servers deployed at roadside units (RSUs) and idle vehicles, elastic computing resources are provided to task vehicles. In this context, vehicles can offload partial tasks from complex applications to VEC servers or roadside units for processing, thereby significantly improving application execution efficiency. However, determining task offloading strategies under various resource and latency constraints remains an unresolved problem [7].

Existing research predominantly focuses on single-agent reinforcement learning or heuristic algorithms for solving offloading strategies, neglecting the policy coupling and competitive relationships inherent in multi-vehicle collaborative decision making, making it difficult for them to adapt to highly dynamic, multi-node, and multi-task concurrent vehicular network environments [8]. Moreover, most existing studies assume that computation results are negligible in size and ideal return links, overlooking the practical risk that high-speed vehicles may have already departed from the RSU communication range before results are generated, leading to result return failure, task invalidation, and surging additional costs [9]. To address this, this paper adopts a task computation result migration strategy: when a vehicle is about to leave the coverage of the current RSU, the system proactively forwards the results to the next adjacent RSU through I2I links; the target RSU then delivers them to the vehicle via I2V links, thereby significantly improving task completion reliability while ensuring real-time performance. Building upon this foundation, we further design the multi-agent reinforcement learning algorithm MATPPO-T to achieve joint optimization of offloading decisions, resource allocation, and result migration. The core contributions of this paper are as follows:

- Model: We propose a multi-layer collaborative computing architecture integrating task vehicles, service vehicles, MEC servers, and cloud computing centers, comprehensively considering task latency, energy consumption, result return failure risk, and vehicle mobility to construct a system model suitable for dynamic traffic environments.
- Problem Formulation: We formalize the multi-vehicle collaborative task offloading problem as a multi-agent Markov decision process (MA-MDP), jointly optimizing task offloading decisions and computing resource allocation, with the objective of minimizing the long-term system task execution cost while balancing dual constraints of latency and energy consumption.
- Algorithm: We propose a multi-agent reinforcement learning algorithm based on improved PPO—multi-agent twin PPO with Transformer (MATPPO-T)—introducing a Transformer-based dual Critic network structure to enhance global state modeling capability and alleviate value overestimation issues, achieving collaborative offloading strategy optimization under the centralized training with decentralized execution paradigm.

## 2. Related Work

The rapid development of vehicular networks has spawned numerous intelligent vehicle models. While these models optimize in-vehicle services and enhance travel convenience, their high-speed mobility characteristics and diverse functional loads also pose significant challenges for the practical application of computation offloading technology, thereby attracting widespread attention in related research directions [10].

Through comprehensive analysis of related literature, we find that most existing studies assume that computation results are negligible in size and ideal return links, overlooking the practical risk that high-speed vehicles may have already departed from RSU communication coverage before results are generated, leading to result return failure, task invalidation, and surging additional costs. In highly dynamic scenarios, such issues become increasingly prominent due to high vehicle density and rapid topology changes. Therefore, vehicle mobility is a factor that cannot be ignored [11], which motivates us to incorporate it into our modeling.

Industry researchers have conducted extensive studies on edge computing task offloading problems and achieved significant progress. They have effectively reduced latency and energy consumption by optimizing task offloading strategies. Among these, some studies have introduced deep reinforcement learning solutions to further enhance the efficiency and performance of task offloading. Zhan et al. [12] proposed a decentralized computation offloading algorithm that addresses the challenge where users in edge computing environments may refuse to disclose their network bandwidth and preference information, combining game theory and deep reinforcement learning methods to achieve strategies where users independently select offloading decisions. Liu et al. [13] significantly improved model adaptability and training efficiency across different vehicular network environments through meta-learning initialization of neural network hyperparameters. Cao et al. [14] proposed a fuzzy inference-based algorithm (F-AOP) to determine current network scenarios (peak or off-peak periods) and adjust optimization objectives accordingly. However, the single-agent perspective fails to characterize the resource competition and policy coupling when multiple vehicles offload simultaneously, causing performance degradation in vehicle-dense scenarios [15]. The existing multi-agent reinforcement learning MAPPO-based offloading methods, such as Kang et al. [16], still adopt the original architecture with a single fully connected Critic network. They do not account for computation result migration and lack mechanisms to prevent overestimation. In highly dynamic vehicular environments, these approaches are prone to value estimation bias and policy oscillation, making it difficult to meet the requirements for reliable offloading. Meanwhile, in high-speed mobility scenarios, single Critic networks exhibit large value estimation variance and severe policy oscillation. Therefore, this paper proposes a multi-agent reinforcement learning algorithm with dual Critic networks, where two independent Critic networks estimate Q-values and take the minimum, directly truncating environmental noise and overestimation errors caused by high-speed mobility, thereby reducing policy gradient variance.

Traditional task offloading strategies mostly rely on static optimization models or heuristic algorithms [17]. Zhu et al. [18] proposed an improved genetic-algorithm-based resource allocation strategy considering delay and energy consumption constraints. Lu et al. [19] presented a novel IoT user-aware task offloading method based on quantum-behaved particle swarm optimization, which effectively reduced time consumption and energy loss by optimizing the location and manner of task offloading. Feng et al. [20] constructed a volunteer-assisted vehicular edge computing model, established a Stackelberg game between volunteers and vehicles, and designed a genetic-algorithm-based fast search algorithm to optimize task offloading and resource allocation. Mohamed et al. [21] proposed a fog computing IoT task offloading and load balancing strategy based on ant colony optimization with response time as the optimization objective. Song et al. [22] divided vehicular tasks into multiple subtasks and proposed an ant colony optimization-based task offloading scheme to minimize the average latency of all tasks in the system. However, static optimization models or heuristic algorithms struggle to cope with complex scenarios in vehicular networks characterized by high vehicle mobility, dynamic network topology changes, and random task requests [23].

In vehicular edge computing networks (VECNs), the global state simultaneously comprises four heterogeneous features: queue load, communication links, task attributes, and computing resources. Traditional fully connected networks merely concatenate these features linearly followed by activation functions, making it difficult to capture high-order coupling relationships such as “the multiplicative effect of task data size and communication distance on transmission latency” and “the matching degree between queue load and task CPU cycles,” leading to large value estimation biases [24]. For example, the MAPPO algorithm adopted by Ning et al. [25] for computation offloading still uses a single fully connected Critic structure, which cannot effectively capture the complex correlations among heterogeneous features and is difficult to adapt to the highly dynamic vehicular environment. In contrast, the multi-head self-attention mechanism of Transformers is inherently adept at capturing such high-order interactions. Moreover, Transformers utilize self-attention to capture long-range dependencies and high-order coupling of variable topologies in a single pass, making them more suitable than CNNs/RNNs for high-dimensional, partially observable, dynamic multi-agent reinforcement learning scenarios [26]. Therefore, this paper introduces a Transformer-based Critic in the algorithm.

Although previous studies have either focused on “single-node single-agent” offloading proportion optimization or achieved static coordination through swarm intelligence and game theory, they generally assume “error-free computation result return with ideal links,” lacking online learning mechanisms for result delivery failure risks caused by high-speed vehicle mobility. Meanwhile, traditional fully connected networks struggle to characterize the dynamic coupling among four heterogeneous features—queue, communication, task, and resources—leading to value estimation bias and policy oscillation. To address this, this paper embeds result migration decisions into the action space through the MATPPO-T framework, utilizes Transformer to encode global heterogeneous states, and employs dual Critic networks with minimum value selection to suppress mobility-induced noise, achieving one-step joint optimization of computation, communication, and forwarding. Under the centralized training with decentralized execution framework, this enables low-latency, low-energy, and highly reliable result delivery in highly dynamic VECN scenarios.

To clarify the distinctions between existing research and this work, Table 1 systematically reviews related literature, presenting a comparative analysis across three aspects: core contributions, research limitations, and the proposed approach of this paper.

## 3. System Model

### 3.1. System Scenario

**Figure 1:**
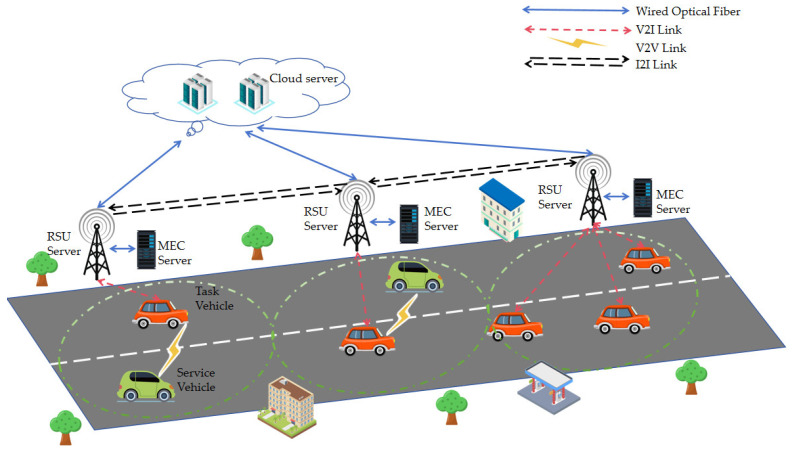
Vehicular Edge Computing Network Scenario.

As shown in Figure 1, we consider a vehicular driving environment consisting of multiple task vehicles traveling at a constant speed v that require task offloading (referred to as vehicle clients), service vehicles parked roadside with available idle resources for providing computing services (referred to as vehicle servers), multiple MEC servers (each equipped with an RSU), and a cloud computing center, collectively forming a vehicular edge computing network (VECN) scenario. The vehicle client can offload computing tasks to the vehicle server or MEC server through V2V and V2I links. The MEC servers are connected to RSUs via wired links, and due to the extremely high communication bandwidth, the transmission time between roadside units and MEC servers can be considered negligible. The cloud computing center is located far from vehicles, and uploading vehicular computation tasks to the cloud center would incur substantial communication time. Therefore, the cloud computing center does not directly undertake task offloading for vehicles; instead, it serves as the control center for the entire region, responsible for monitoring vehicle distribution within the area and performing resource allocation and adjustment to optimize overall system operational efficiency [27]. To address the issue of failed backhaul of computation results caused by vehicle movement, the system supports a result migration mechanism between RSUs. It forwards unfinished backhaul results to the next adjacent RSU through an I2I link, ensuring reliable delivery of task results.

In this scheme, vehicle task arrivals follow a Poisson distribution [28], and each vehicle can continuously generate multiple computation-intensive or latency-sensitive tasks, such as image recognition, natural language processing, intelligent navigation, and augmented reality [29], with each task’s offloading decision made independently. Moreover, vehicles can only transmit data when located within the coverage range of an MEC server. The diameter of each MEC coverage range is set to D. L denotes the distance between the RSU coverage range and the rear of the vehicle when the vehicle initiates a computation offloading task. The residence time t of the vehicle within the current roadside unit coverage range, starting from when the vehicle begins computation task offloading, is:

(1) $$t=\frac{D-L}{v}$$

The set of vehicle clients is denoted as $N=\left\{1,\;2,\;...,\;n\right\}$, and the set of vehicle servers is denoted as $M=\left\{1,\;2,\;...,m\right\}$. We use a triplet $Task\left(i\right)=\left(D_{i},\;C_{i},\;T_{max}\right)$ to represent the information of a task requiring offloading, where $D_{i}$ denotes the data size for transmission, $C_{i}$ denotes the number of CPU cycles required to complete the task, and $T_{max}$ represents the maximum tolerable execution time for the task. This study adopts a binary task offloading strategy, meaning that a task must be executed as an intact computational unit either locally, on an MEC server, or on a service vehicle [30].

### 3.2. Communication Model

In a noisy and bandwidth-constrained channel environment, according to Shannon’s theorem [31], the theoretical upper limit of the error-free transmission rate that data can achieve can be expressed as:

(2) $$C=B\times \log_{2}(1+\frac{S}{N})$$

where C is the channel capacity (maximum transmission rate), B is the channel bandwidth, S is the average signal power, and N is the average noise power.

This paper employs the Shannon capacity formula as the foundation for channel modeling, introducing the following simplifying assumptions to ensure analytical tractability: (1) an additive white Gaussian noise (AWGN) channel model is adopted; (2) it is assumed that the coding block length is sufficiently long to experience all fading states, i.e., the ergodic channel capacity is applicable; (3) the channel is quasi-static within a time slot, meaning the channel coherence time exceeds the task transmission duration; (4) perfect channel state information is available at the receiver. These assumptions represent standard simplifications in the field of vehicular edge computing and computation offloading. For the scenarios in this paper, the online learning mechanism of the proposed reinforcement learning algorithm can effectively adapt to the dynamic variations of the channel.

#### 3.2.1. V2I Communication Model

The data transmission rate between vehicle clients and MEC servers can be expressed according to the Shannon formula as:

(3) $$R_{n,rsu}=B\log_{2}(1+\frac{p_{n}h_{k}}{N_{0}})$$

where $R_{n,rsu}$ denotes the data transmission rate between the vehicle client and the MEC server, B denotes the channel bandwidth, $N_{0}$ is the Gaussian noise power within the channel, $P_{n}$ is the transmit power of the vehicle client, and $h_{k}$ denotes the channel gain. The channel gain $h_{k}=G_{0}d_{k}^{-\alpha }g_{k}$ incorporates path loss and Rayleigh fading, assumed quasi-static within each time slot.

#### 3.2.2. V2V Communication Model

The data transmission rate between vehicle clients and vehicle servers can be expressed according to the Shannon formula as:

(4) $$R_{n,m}=B\log_{2}(1+\frac{p_{n}h_{k}}{N_{0}})$$

where $R_{n,m}$ denotes the data transmission rate between the vehicle client and the vehicle server, B denotes the channel bandwidth, $N_{0}$ is the Gaussian noise power within the channel, $P_{n}$ is the transmit power of the vehicle client, and $h_{k}$ denotes the channel gain. V2V channels exhibit higher dynamics due to relative motion between vehicles; rate adaptation mechanisms would be required in practice.

#### 3.2.3. I2I Communication Model

In this VECN system, we consider the communication links between adjacent RSUs for transmitting computation results. When vehicle speed is excessively high, collaborative transmission of computation results between adjacent RSUs needs to be considered. In this case, assuming that ${RSU}_{j}$ and ${RSU}_{k}$ are adjacent and their communication coverage areas border each other without overlapping regions, the data transmission rate between ${RSU}_{j}$ and ${RSU}_{k}$ can be expressed as:

(5) $$R_{j,k}=B\log_{2}(1+\frac{p_{rsu}h_{k}}{N_{0}})$$

where $R_{j,k}$ denotes the data transmission rate between MEC server and MEC server, B denotes the channel bandwidth, $N_{0}$ is the Gaussian noise power within the channel, $P_{rsu}$ is the transmit power of the MEC server, and $h_{k}$ denotes the channel gain. I2I links are stable wired/backhaul connections where mobility effects are negligible.

### 3.3. Computation Model

Vehicle clients have three task offloading methods: local computing, offloading to vehicle servers, and offloading to RSUs equipped with MEC servers. In real-world vehicular network applications, there are many urgent tasks such as traffic accidents, road congestion, and emergency rescue that require real-time feedback; therefore, the return of task results cannot be neglected. The latency and energy consumption of computation result return links are key metrics that require careful consideration [32]. Thus, we define the relationship between computation result size and input raw data size as:

(6) $$D_{i,out}=\frac{D_{i}}{\eta_{task}}$$

where $\eta_{task}$ denotes the task data processing coefficient, with a value of 100. This coefficient is a universal value for the ratio of the result data volume to the input data volume in the unloading task of vehicle networking computing [33]. Considering that computation result return and task offloading can reuse the same transmission channel in different communication models, it is reasonable to assume that their transmission rates are equal.

#### 3.3.1. Local Computing Model

When the vehicle client has sufficient computing resources, it can choose to process the computation task locally only. When the computation task is executed locally, the computation offloading latency depends only on the computing capability (CPU cycles) $f_{n}$ of the vehicle client’s local terminal [34]; thus, the latency can be expressed as:

(7) $$T_{loc}=\frac{C_{i}}{f_{n}}$$

When the computation task is executed locally, energy is consumed only during the execution of the computation task; therefore, the energy consumed for executing the computation task locally is:

(8) $$E_{loc}=k_{n}{\left(f_{n}\right)}^{2}o_{n}$$

where $k_{n}$ denotes the effective switching capacitance, whose magnitude depends on the CPU chip architecture, and $o_{n}$ is the unit resource cost.

#### 3.3.2. Offloading to Vehicle Server Computing Model

Due to the binary offloading strategy adopted for computation tasks, partial offloading is not considered. At any given moment, each vehicle client can select one vehicle server to execute the computation task. If the vehicle server has additional idle resources at that moment, it can serve multiple vehicle clients [35]. However, due to the limited computing resources of vehicle servers, multiple tasks may arrive simultaneously and need to queue for service.

Therefore, in the process of offloading computation tasks to vehicle servers, the total time comprises four main components: the time required to transmit the computation task from the vehicle client to the vehicle server, the queuing waiting time for task execution, the computation execution time on the vehicle server, and the time required for the vehicle server to return the task result to the vehicle client, which are respectively:

(9) $$T_{trans,up}^{m}=\frac{D_{i}}{R_{n,m}}$$

(10) $$T_{\mathrm{queue}\;}^{m}=\frac{\varphi_{m}}{\mu_{m}\left(\mu_{m}-\varphi_{m}\right)}$$

(11) $$T_{comp}^{m}=\frac{C_{i}}{f_{m}}$$

(12) $$T_{trans,back}^{m}=\frac{D_{i,out}}{R_{n,m}}$$

where $\mu_{m}$ is the actual number of tasks arriving at the vehicle server per unit time, $\varphi_{m}=\frac{f_{m}}{C_{avg}}$, $C_{avg}$ is the average number of CPU cycles of all tasks offloaded to this server per unit time, and $f_{m}$ denotes the computing capability (CPU cycles) of the vehicle server that can provide computing services.

The total time required for the vehicle client to offload the task to the vehicle server is:

(13) $$T_{total}^{m}=T_{trans,up}^{m}+T_{queue}^{m}+T_{comp}^{m}+T_{trans,back}^{m}$$

The energy consumption for transmitting the computation task from the vehicle client to the vehicle server, the energy consumption for executing the computation task on the vehicle server, and the energy consumption for returning the task result from the vehicle server to the vehicle client are respectively:

(14) $$E_{trans,up}^{m}=p_{n}T_{trans,up}^{m}=p_{n}\frac{D_{i}}{R_{n,m}}$$

(15) $$E_{comp}^{m}=k_{m}{\left(f_{m}\right)}^{2}o_{m}$$

(16) $$E_{trans,back}^{m}=p_{m}T_{trans,back}^{m}=p_{m}\frac{D_{i,out}}{R_{n,m}}$$

where $k_{m}$ denotes the effective switching capacitance of the vehicle server, whose magnitude depends on the CPU chip architecture, $o_{m}$ is the unit resource cost, $f_{m}$ is the computing capability of the vehicle server (CPU cycles), and $p_{m}$ is the transmit power of the vehicle server.

Therefore, the total energy consumption for offloading the computation task to the vehicle server is:

(17) $$E_{total}^{m}=E_{trans,up}^{m}+E_{comp}^{m}+E_{trans,back}^{m}$$

#### 3.3.3. Offloading to RSU Computing Model

To address the core contradiction of result return failure caused by task vehicles driving out of RSU coverage, this system considers the task result forwarding mechanism between adjacent RSUs and the RSU↔vehicle client result delivery mechanism. Therefore, in the process of offloading computation tasks to an RSU, the total time comprises five main components: the time required to transmit the computation task from the vehicle client to the RSU, the queuing waiting time for task execution, the computation execution time on the RSU, the transmission time required for the task result between adjacent RSUs, and the time required for the RSU to return the task result to the vehicle client, which are respectively:

(18) $$T_{trans,up}^{rsu}=\frac{D_{i}}{R_{n,rsu}}$$

(19) $$T_{\mathrm{queue}\;}^{rsu}=\frac{\varphi_{rsu}}{\mu_{rsu}\left(\mu_{rsu}-\varphi_{rsu}\right)}$$

(20) $$T_{comp}^{rsu}=\frac{C_{i}}{f_{rsu}}$$

(21) $$T_{trans,I2I}^{rsu}=\frac{D_{i,out}}{R_{j,k}}$$

(22) $$T_{trans,back}^{rsu}=\frac{D_{i,out}}{R_{n,rsu}}$$

where $\mu_{rsu}$ is the actual number of tasks arriving at the RSU per unit time, $\varphi_{rsu}=\frac{f_{rsu}}{C_{avg}}$, $C_{avg}$ is the average number of CPU cycles of all tasks offloaded to this server per unit time, and $f_{rsu}$ denotes the computing capability (CPU cycles) of the RSU that can provide computing services.

Therefore, the total time required for the vehicle client to offload the task to the RSU is:

(23) $$T_{total}^{rsu}=T_{trans,up}^{rsu}+T_{queue}^{rsu}+T_{comp}^{rsu}+T_{trans,I2I}^{rsu}+T_{trans,back}^{rsu}$$

where, when the vehicle moves at low speed and the task result has already been returned to the vehicle client before driving out of the RSU coverage, the task result does not need to be transmitted between adjacent RSUs, and its cross-RSU transmission time $T_{trans,I2I}^{rsu}\;$ can be regarded as 0. When the vehicle moves at high speed and the task result has not been returned to the vehicle client when driving out of the RSU coverage, i.e., $T_{trans,up}^{rsu}+T_{queue}^{rsu}+T_{comp}^{rsu}+T_{trans,back}^{rsu}>t$, the cross-RSU transmission time needs to be calculated according to Equation (20).

The energy consumption for transmitting the computation task from the vehicle client to the RSU, the energy consumption for executing the computation task on the RSU, and the energy consumption for returning the task result from the RSU to the vehicle client are respectively:

(24) $$E_{trans,up}^{rsu}=p_{n}T_{trans,up}^{rsu}=p_{n}\frac{D_{i}}{R_{n,rsu}}$$

(25) $$E_{comp}^{rsu}=p_{r}T_{comp}^{rsu}=p_{r}\frac{C_{i}}{f_{rsu}}$$

(26) $$E_{trans,back}^{rsu}=p_{rsu}T_{trans,back}^{rsu}=p_{rsu}\frac{D_{i,out}}{R_{n,rsu}}$$

where $p_{r}$ is the computing power of the MEC server.

Therefore, the total energy consumption for offloading the computation task to the RSU is:

(27) $$E_{total}^{rsu}=E_{trans,up}^{rsu}+E_{comp}^{rsu}+E_{trans,back}^{rsu}$$

where, since the RSU is continuously powered by a fixed power supply and the power consumption of the wired backbone links between them is unrelated to the vehicle client, the total energy consumption for offloading the computation task to the RSU does not include the energy consumption for transmitting the task result between adjacent RSUs.

Table 2 lists the key symbols used in Chapter 3 and their physical meanings:

## 4. Problem Definition

This paper assumes that, during the simulation process, vehicles continuously generate multiple tasks. Let w be the number of tasks completed by the vehicle at a certain moment, where x is the number of tasks computed locally, y is the number of tasks offloaded to RSU for execution, and z is the number of tasks offloaded to vehicle servers for execution, satisfying w=x+y+z.

Define total latency as T, total energy consumption as E, and the total cost of the joint offloading system as H:

(28) $$T=\sum_{i=0}^{x}T_{loc}+\sum_{i=0}^{y}T_{total}^{m}+\sum_{i=0}^{z}T_{total}^{rsu}$$

(29) $$E=\sum_{i=0}^{x}E_{loc}+\sum_{i=0}^{y}E_{total}^{m}+\sum_{i=0}^{z}E_{total}^{rsu}$$

(30) H=αT+βE

where α and β are the latency weight factor and energy consumption weight factor, respectively, satisfying 0≤α≤1, 0≤β≤1, and α+β=1. The differentiated values of latency weight factor and energy consumption weight factor correspond to the heterogeneous requirements of vehicular tasks in terms of latency and energy consumption; for personalized requirements of different types of tasks, the decision-making process can be optimized by selecting appropriate weight parameters. The objective of this paper is to minimize the total cost of the joint offloading system while satisfying the constraints of maximum tolerable latency $T_{max}$ and resources, modeled as follows:

(31) $$\min\sum_{i=0}^{n}H=\min\sum_{i=0}^{n}\left[\alpha T+\beta E\right]$$

(32) $$\left\{\begin{array}{l} C1:T_{loc}<T_{\max}\\ C2:T_{total}^{m}<T_{\max}\\ C3:T_{total}^{rsu}<T_{\max}\\ C4:w=x+y+z\\ C5:0<f_{rsu}<f_{rsu}^{\max}\\ C6:0<f_{m}<f_{m}^{\max} \end{array}\right.$$

where C1, C2, and C3 ensure that the task latency requirements of vehicle clients are satisfied; C4 indicates that the sum of the number of tasks computed locally (x), offloaded to RSU for execution (y), and offloaded to vehicle servers for execution (z) equals the total number of currently executed tasks; C5 specifies that, if the vehicle client offloads the computation task to an RSU, the computing resources allocated by the RSU should be greater than 0 and less than the resource upper limit; C6 specifies that, if the vehicle client offloads the computation task to a vehicle server, the computing resources allocated by the vehicle server should be greater than 0 and less than the resource upper limit.

## 5. Algorithm Design

### 5.1. Multi-Agent Markov Decision Process

Multi-agent Markov decision process (MA-MDP) is an extension of the Markov decision process (MDP) to multi-agent scenarios [36], where the environment state still maintains the Markov property. All agents select actions based on their respective observations in the same time slot, and the joint actions collectively drive the transition to the next state and determine the shared global immediate reward. The payoff of each agent depends not only on its own strategy but also on the strategies of other agents, forming a coupled decision-making problem of “joint state—joint action—global reward.”

This paper uses a sextuple $\left(U,S,A,P,R,\gamma \right)$ to describe the multi-agent Markov decision process model. $U=\left\{1,\;2,\;...,\;u\right\}$ represents the set of agent quantities, S is the environment state space, A represents the joint action space of the intelligent agents, P is the state transition function of the environment, R is the agent reward function, and γ is the reward discount factor.

#### 5.1.1. State Space

At time slot k, let $S_{k}=\left\{Q_{h},D_{n},F_{n},C_{h}\right\}$ denote the environment state space, where

(33) $$Q_{h}=\left\{q_{1,k},q_{2.k},\ldots ,q_{m,k}\right\}$$

(34) $$D_{n}=\left\{d_{1,k},d_{2,k},\ldots ,d_{m,k}\right\}$$

(35) $$F_{n}=\left\{f_{1,k},f_{2,k},\ldots ,f_{m,k}\right\}$$

(36) $$C_{h}=\left\{c_{1,k},c_{2,k},\ldots ,c_{m,k}\right\}$$

where $q_{i,k}$ denotes the computing queue load of the i-th service node in time slot k; $d_{j,k}$ denotes the transmission data size of the j-th task of the vehicle client in time slot k; $f_{j,k}$ denotes the computing requirement (CPU cycles) of the j-th task of the vehicle client in time slot k; and $c_{i,k}$ denotes the available computing resources of the i-th service node in time slot k.

#### 5.1.2. Observation Space

This study is based on the assumption of a partially observable environment, where each agent can only obtain its own and surrounding local information. Therefore, at time slot k, the observation space of agent u is:

(37) $$O_{u,k}=\left\{q_{u,k},d_{u,k},f_{u,k},c_{u,k}\right\}$$

#### 5.1.3. Action Space

At time slot k, each agent outputs an action based on the current observation information as the offloading strategy adopted in the current time slot. To achieve joint optimization of task offloading, resource allocation, and result migration, this paper defines the action space as a joint action comprising three decision components. The action space can be expressed as:

(38) $$A=a_{n,k}=(x_{n,k},f_{n,k}^{alloc},m_{n,k})$$

The action space consists of three dimensions, where the offloading decision $x_{n,k}\in \{0,1,2\}$ determines the task execution location (0 for local, 1 for RSU, and 2 for service vehicle); the resource allocation decision $\;f_{n,k}^{alloc}$ represents the proportion of CPU computing resources allocated by the target service node for the current task, satisfying the constraint $\;\;0<f_{n,k}^{alloc}\leq f_{max}$, $f_{max}$ represents the maximum computing capability of the RSU or vehicle server; and the result migration decision $m_{n,k}\in \{0,1\}$ determines whether to proactively migrate the result to the next RSU (0 for direct return, and 1 for pre-migration).

#### 5.1.4. Reward Function

In multi-agent reinforcement learning, reward design needs to guide the system optimization objective. This paper uses the negative value of the objective function as the reward value, and the reward is defined as:

(39) R=−H=−(αT+βE)

### 5.2. MATPPO-T Algorithm

The objective of this paper is to optimize the edge computing offloading strategy in vehicular networks, maximizing system utility by determining the optimal offloading proportion for all vehicles after task generation in each time slot. In the constructed model, all vehicle clients face multiple offloading proportion choices, which interact with each other, constituting a complex NP-hard decision problem with high solution difficulty [37]. To address this complex decision problem, this section conducts an in-depth study of the deep reinforcement learning algorithm: MAPPO algorithm [38], and improves the MAPPO algorithm for the research problem. By optimizing algorithm performance, we strive to find a more efficient and precise offloading strategy to achieve maximization of system utility.

Although the MAPPO algorithm demonstrates superior performance over traditional decentralized reinforcement learning in multi-agent collaborative decision-making scenarios through the “independent policy update + shared central Critic network” framework [39], when directly applied to vehicular network edge computing offloading tasks, it still faces the following core adaptability deficiencies, making it difficult to meet requirements for decision accuracy, system stability, and environmental adaptability.

MATPPO-T is further optimized based on MAPPO, enhancing the algorithm’s scenario adaptability through improved network architecture. The specific improvements are as follows:

(1) Transformer-based Critic Network Structure Improvement: The shared central Critic network in MAPPO mostly adopts simple fully connected networks, which merely concatenate global state features through linear transformations and activation functions. This makes it difficult to capture deep non-linear associations among heterogeneous features such as queue load, communication distance, task requirements, and resource status (e.g., “the coupling effect of task data size and communication distance on transmission latency,” “the matching degree association between queue load and task computing requirements”), resulting in limited value estimation accuracy. MATPPO-T reconstructs the central Critic network into a Transformer-based architecture. Through a feature tokenization module, the global state is semantically decomposed into four types of tokens: “queue load features,” “communication features,” “task features,” and “resource features.” Then, a multi-head attention encoder is used to capture complex associations among features of different dimensions. Finally, position encoding and attention pooling are combined to enhance key information, significantly improving the feature expression capability of global states and the precision of value estimation.
(2) Dual Critic Networks: MAPPO relies on a single-path central Critic for value estimation, lacking fault tolerance capabilities for environmental noise and dynamic disturbances. In vehicular network environments, dynamic factors such as communication link quality fluctuations caused by vehicle mobility, load variations triggered by Poisson-distributed task arrivals, and result return link changes due to RSU coverage handovers can easily cause value estimation oscillations in single Critic networks, thereby affecting the stability of policy updates. MATPPO-T employs two independent Critic networks for value estimation, selecting the smaller value when computing the target value. This effectively alleviates the problem of value overestimation and significantly improves algorithm performance.

**Figure 2:**
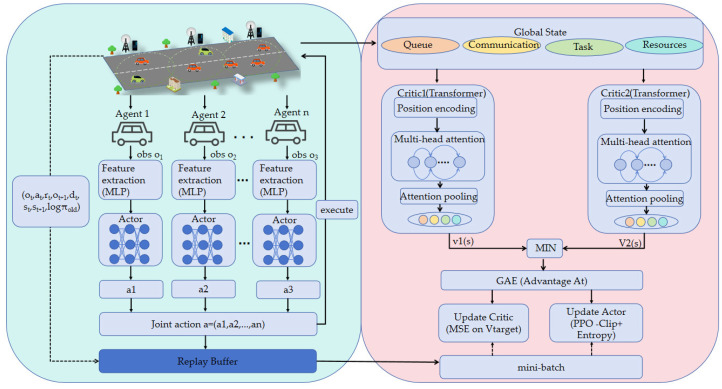
Algorithm Structure of MATPPO-T.

The algorithm structure design of MATPPO-T is shown in Figure 2. The algorithm adopts a centralized training and decentralized execution framework: each agent (vehicle) generates local offloading decisions through an independent Actor network; the global state is input into a dual Transformer–Critic network after feature splitting, where value estimation is performed separately and the smaller value is taken to suppress overestimation; multi-head self-attention is utilized to capture the high-order coupling relationships among four types of heterogeneous features: queue, communication, task, and resource; finally, PPO clip loss and advantage function are combined to complete policy optimization, achieving joint optimization of offloading decisions, resource allocation, and result migration.

The multi-agent reinforcement learning algorithm adopted in this paper is based on the proximal policy optimization (PPO) framework, combining Transformer global state encoding with the twin Critic mechanism to form a centralized training with decentralized Execution (CTDE) paradigm suitable for distributed decision-making scenarios [40]. Each agent is equipped with an independent Actor network to achieve decentralized decision making. This network adopts an MLP structure, first mapping the input observation vector $s_{i}\in R^{d_{obs}}$ to a hidden vector through a feature extraction module:

(40) $$f_{i}=MLP(s_{i})$$

Subsequently, a multi-head output structure is employed to generate the joint action distribution: the offloading head outputs the probability distribution of the offloading decision $x_{n}$ through a softmax layer, the resource head outputs the resource allocation proportion $f_{n}^{alloc}$ through a sigmoid layer, and the migration head outputs the probability distribution of the result migration decision $m_{n}$ through a softmax layer:

(41) $$\pi_{i}^{x}(\left.x_{n}\right|s_{i})=soft\max(MLP_{x}(f_{i}))\;\pi_{i}^{f}(\left.f_{n}^{alloc}\right|s_{i},x_{n})=sigmoid(MLP_{f}(f_{i}))\;\pi_{i}^{m}(\left.m_{n}\right|s_{i},x_{n})=soft\max(MLP_{m}(f_{i}))$$

The joint probability of the *i*-th agent selecting the composite action $a_{i}=(x_{i},f_{i}^{alloc},m_{i})$ is the product of the three:

(42) $$\pi_{i}(\left.a_{i}\right|s_{i})=\pi_{i}^{x}(x_{i}|s_{i})\cdot \pi_{i}^{f}(f_{i}^{alloc}|s_{i},x_{i})\cdot \pi_{i}^{m}(m_{i}|s_{i},x_{i})$$

The global value function is centrally estimated by dual Critic networks, with each Critic network employing Transformer to encode the global state: first, the global state $S=\left[s_{1},s_{2},\ldots ,s_{N}\right]\in R^{N\cdot d_{obs}}$ is semantically segmented into four types of tokens—queue state, device attributes, task features, and computing resources—and embeddings are generated through linear projections respectively:

(43) $$t_{queue}=\mathrm{Pr}oj_{queue}(S_{queue})$$

(44) $$t_{veh}=\mathrm{Pr}oj_{veh}(S_{veh})$$

(45) $$t_{task}=\mathrm{Pr}oj_{task}(S_{task})$$

(46) $$t_{mips}=\mathrm{Pr}oj_{mips}(S_{mips})$$

Position encoding is then added and fed into the Transformer encoder, yielding:

(47) $$z_{1},\ldots ,z_{4}=Transformer\left(\left\{t_{queue}+p_{1},\ldots ,t_{mips}+p_{4}\right\}\right)$$

Finally, global features are generated through attention pooling, and the value estimation is output:

(48) $$v(S)=MLP(AttnPool(z_{1},\ldots ,z_{4}))$$

To alleviate the problem of value overestimation, the algorithm adopts a dual Critic network mechanism, where the target value takes the minimum of the outputs from two independent Critic networks $Q_{1}$ and $Q_{2}$, i.e.,:

(49) $$Q_{t\arg et}(S,a)=\min(Q_{1}(S,a),Q_{2}(S,a))$$

During training, the mean squared error losses of the two networks are minimized respectively:

(50) $$L_{Q_{1}}=E[{\left(Q_{1}(S,a)-y_{r}\right)}^{2}]$$

(51) $$L_{Q_{2}}=E[{\left(Q_{2}(S,a)-y_{r}\right)}^{2}]$$

where the target value is:

(52) $$y_{r}=r+\gamma (1-d)Q_{t\arg et}(S^{\prime },a^{\prime })$$

where r is the immediate reward, d is the termination flag, and γ is the discount factor.

The Actor network achieves policy optimization through PPO’s clipped loss (CLIP), ensuring controllable update magnitude: first, the importance sampling ratio is calculated:

(53) $$r_{t}=\frac{\pi_{\theta }(\left.a_{t}\right|s_{t})}{\pi_{\theta \;old}(\left.a_{t}\right|s_{t})}$$

Then the clipped loss function is constructed:

(54) $$L=-E[min\left(r_{t}A_{t},clip\left(r_{t},1-\epsilon ,1+\epsilon \right)A_{t}\right)-\eta H\left(\pi_{\theta }\right)]$$

where $A_{t}$ is the generalized advantage estimation (GAE), ϵ is the clipping coefficient, η is the entropy regularization coefficient, and H(π) is the entropy of the action distribution.

The complete training process of the algorithm is as follows: first, agents interact with the environment based on the current policy and store trajectory data $\left(s_{i},a_{i},r_{i},d_{i},S\right)$; then, advantage values $A_{t}=\sum_{k=0}^{T-t-1}(\gamma \lambda {)}^{k}\delta_{t+k}$ are estimated through GAE (where $\delta_{t}=r_{t}+\gamma V(s_{t+1})-V(S_{t})$); finally, data is randomly sampled to alternately optimize the Critic (minimizing $L_{Q_{1}}+L_{Q_{2}}$) and the Actor (minimizing $L_{\pi }$), with update stability controlled through gradient clipping.

Algorithm 1 summarizes the offloading strategy and computing the resource allocation algorithm based on MATPPO-T.
**Algorithm 1:** MATPPO-T Algorithm1: Input: Global state $s_{t}$, local observation $o_{t}^{i}$, maximum number of episodes E, experience replay buffer D, discount factor γ, clipping coefficient ϵ, entropy coefficient η
2: Output: Trained policy network $\pi_{\theta }$ and dual Transformer value networks $Q_{\phi 1}$, $Q_{\phi 2}$
3: Initialize: Actor network $\pi_{\theta }^{i}(MLP+Soft\max)$, dual Transformer Critic networks $Q_{\phi 1}$, $Q_{\phi 2}$, and target networks4:
Initialize experience replay buffer D
$,\;\mathrm{global}\;\mathrm{state}\;\mathrm{encoder}\;{Transformer}_{global}$
5: for episode = 1 to E do6:            $\mathrm{Reset}\;\mathrm{environment},\;\mathrm{obtain}\;\mathrm{initial}\;\mathrm{observation}\;o_{0}^{i}$
7:            for each time step t do8:                   Each agent i$\;\mathrm{selects}\;\mathrm{action}\;a_{i,t}=(x_{i,t},f_{i,t}^{alloc},m_{i,t})$ $\mathrm{according}\;\mathrm{to}\;\pi_{\theta }^{i}(o_{t}^{i})$
9:                   $\mathrm{Execute}\;\mathrm{joint}\;\mathrm{action}a_{t}=\left\{a_{1,t},\ldots ,a_{N,t}\right\}$$,\;\mathrm{obtain}\;\mathrm{reward}\;r_{t}$$\;\mathrm{and}\;\mathrm{next}\;\mathrm{state}\;S_{t+1}$
10:                 $\mathrm{Construct}\;\mathrm{global}\;\mathrm{state}\;S_{t}=concat\left\{o_{t}^{1},\ldots ,o_{t}^{N}\right\}$, input to Transformer encoder11:                 $\mathrm{Store}\;\mathrm{transition}\;\mathrm{sample}\;(S_{t},a_{t},r_{t},S_{t+1})$ to D
12:                 if update cycle then13:                        Sample a batch of data from D
14:                       $\;\mathrm{Compute}\;\mathrm{target}\;\mathrm{value}\;\mathrm{using}\;\mathrm{dual}\;\mathrm{Critic}:\;y=r_{t}+\gamma \min_{j=1,2}Q_{\varphi_{j}}(S_{t+1},a_{t+1})$
15:                       $\mathrm{Update}\;\mathrm{Critic}:\;\mathrm{Minimize}\;\mathrm{MSE}\;\mathrm{loss}\;L_{Q}=\sum_{i=1}^{2}{\left(Q_{\phi i}-y\right)}^{2}$
16:                       $\;\mathrm{Compute}\;\mathrm{advantage}\;A_{t}$ using GAE17:                       $\mathrm{Update}\;\mathrm{Actor}:\;\mathrm{Minimize}\;\mathrm{PPO}\;\mathrm{clipped}\;\mathrm{loss}\;L_{\pi }=-E[min(r_{t}A_{t},clip(r_{t},1-\epsilon ,1+\epsilon )A_{t}]-\eta H(\pi )$
18:                       Soft update target networks19:                   end if20:            end for21: end for22: $\mathrm{return}\;\pi_{\theta }$$,\;Q_{\phi 1}$, $Q_{\phi 2}$


## 6. Experimental Results and Analysis

### 6.1. Experimental Environment Setup

This section first establishes the experimental simulation environment and clarifies parameter configurations. We simulate various task offloading schemes based on the PyTorch 2.8.0 framework on a 2.70 GHz Intel Core i7 processor (manufactured by Intel Corporation, Santa Clara, CA, USA) and then conduct systematic comparative analysis of various schemes and algorithms under different experimental scenarios.

The experimental results validate the effectiveness of the offloading strategy based on the MATPPO-T algorithm, with relevant conclusions supported by the statistical results of experimental simulation parameters in Table 3.

This paper selects typical offloading strategies in recent years as comparison benchmarks, including the PPO algorithm based on deep reinforcement learning [41] and the mainstream multi-agent reinforcement learning framework MAPPO, while additionally introducing two boundary strategies: all-local execution (Local) and all-offloading to RSU (RSU). The core purpose of introducing these two boundary strategies is to precisely delineate the advantage boundaries and applicable scope of the proposed method compared to mainstream algorithms under specific performance metrics and task scenarios, thereby more intuitively highlighting the effectiveness and superiority of the algorithm.

To ensure the statistical reliability of experimental results, all simulation experiments were independently repeated 10 times under identical parameters, with the results presented as mean values. The standard deviation of all metrics was controlled within 3% of the mean, demonstrating the strong stability of the proposed algorithm and the credibility of the experimental results.

To ensure stable training and optimal performance of the proposed MATPPO-T algorithm, the key hyperparameters used in this work are listed in Table 4:

### 6.2. Experimental Results Analysis

**Figure 3:**
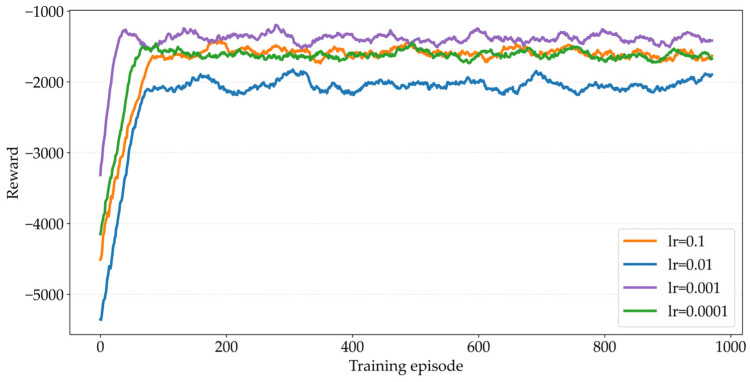
Comparison of Different Learning Rates.

**Figure 4:**
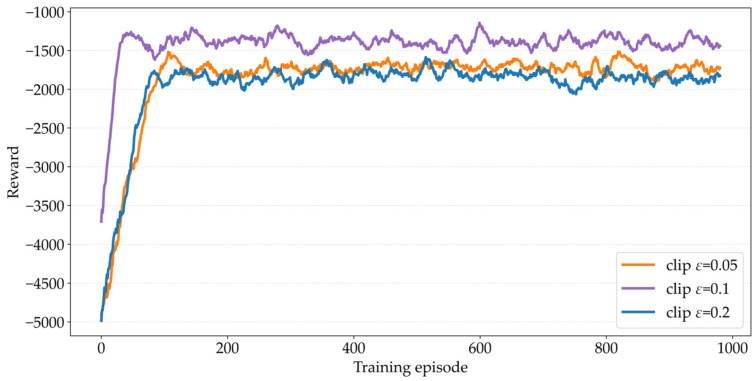
Comparison of Different Clip Thresholds.

**Figure 5:**
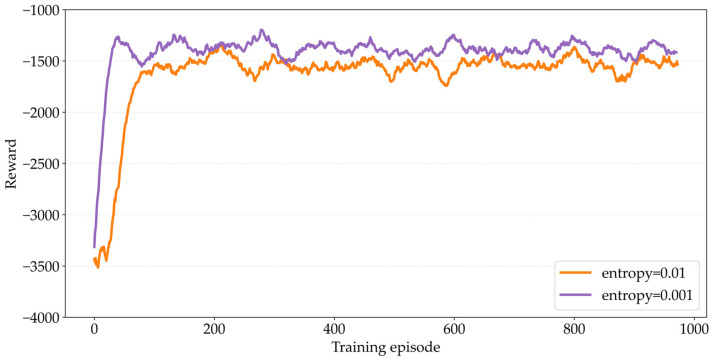
Comparison of Different Entropy Regularization Coefficients.

The multi-agent proximal policy optimization (MAPPO) algorithm has become one of the mainstream methods for solving multi-agent collaborative problems. However, in dynamically changing environments, its performance is susceptible to interference from factors such as uncertainty in environmental states and coupling relationships among agent interactions, thereby exhibiting significant scenario dependency. In view of this, to achieve optimal performance of the MATPPO-T algorithm in target application scenarios, systematic debugging and optimization of its core parameters are crucial. Therefore, this paper compares the impact of different parameters on the proposed scheme, as shown in Figure 3, Figure 4 and Figure 5.

The learning rate directly determines the step size of policy updates, and its reasonable value significantly affects whether the algorithm can quickly converge to the optimal solution or whether oscillation and divergence issues occur [42]. Figure 3 describes the impact of different learning rates in the Actor and Critic networks on experimental performance. When lr=0.001, the agent obtains the maximum reward. Through comparison, it can be seen that the performance is optimal at this point, with the curve approaching convergence after approximately 40 iterations; when lr=0.1 and lr=0.0001, the overall upward trend of the curve is the most gradual, with the reward value stabilizing at only around −2200 even after 1000 training episodes, and the convergence speed is relatively slower. When lr=0.01, the agent obtains the lowest reward value. The performance of the MATPPO-T algorithm can be optimized when the Actor and Critic network learning rates are set to 0.001; therefore, in subsequent experiments, lr=0.001 is adopted as the learning rate for the Actor and Critic networks.

To ensure training stability and avoid model oscillation or divergence caused by excessive policy update magnitude, the MAPPO algorithm introduces a clipping (clip) mechanism, whose core function is to constrain the probability distribution difference between the new and old policies. In the policy gradient computation phase, the algorithm first evaluates the deviation degree between the original and new policies in terms of probability distribution, then employs the clip mechanism to ensure this deviation does not exceed a preset threshold limit. Figure 4 compares the performance of the MATPPO-T algorithm under different clip thresholds. Through comparison, it can be seen that, when clip ϵ=0.1, the convergence is fastest, with the curve approaching convergence after approximately 40 iterations; when clip ϵ=0.05 and clip ϵ=0.2, the convergence speed is relatively slower with slightly lower rewards. The performance with clip ϵ=0.1 is relatively better in later stages with a slightly higher reward upper limit. Therefore, in subsequent experiments, clip ϵ=0.1 is adopted as the threshold for the clipping mechanism.

The MATPPO-T algorithm in this paper enhances exploration by introducing a policy entropy exploration mechanism to balance the agent’s “action exploration” and “experience exploitation.” The value of the entropy regularization coefficient directly affects policy characteristics [43]: the larger the coefficient, the more significant the penalty on policy entropy, and the enhanced policy randomness can promote the agent to explore more actions and avoid premature convergence; the smaller the coefficient (approaching 0), the more deterministic the policy becomes, which can quickly reuse optimal actions to improve convergence speed but is prone to falling into local suboptima. Figure 5 compares the performance of the MATPPO-T algorithm under different entropy regularization coefficients. It can be observed that, when entropy=0.001, the convergence speed is relatively faster, and the reward is also higher than when entropy=0.01. Therefore, in subsequent experiments, entropy=0.001 is adopted as the entropy regularization coefficient for the policy entropy exploration mechanism.

Based on the tuning experiment in Figure 3, Figure 4 and Figure 5, the degree of influence of each hyperparameter on the performance of MATPPO-T is as follows: learning rate > clip threshold > entropy coefficient. Deviation of learning rate from the optimal value of 0.001 by one order of magnitude can lead to a decrease of 18–22% in the final reward and convergence failure. When the clip threshold deviates from 0.1, the reward decreases by 8–12%. The entropy coefficient has the least impact and only decreases by 5–8% when deviating. Actual deployment should prioritize fine-tuning the learning rate.

**Figure 6:**
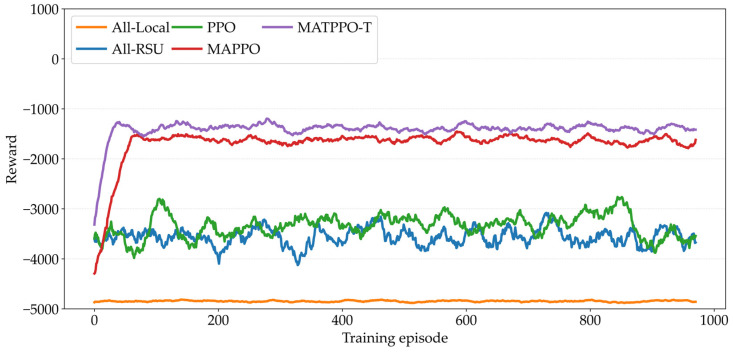
Comparison of training reward curves for various task offloading schemes.

Figure 6 compares the changes in average reward per episode during training for five schemes: all-local, all-RSU, PPO, MAPPO, and the proposed MATPPO-T. A higher reward value indicates a lower weighted total cost of system latency and energy consumption and a more optimal strategy. It can be observed that all-local computing and all-offloading to RSU computing exhibit slow convergence or even failure to converge due to their lack of dynamic task allocation capabilities and difficulty in adapting to the dynamic characteristics of vehicular networks. The performance of PPO and MAPPO gradually improves, but the convergence speed is limited; the proposed MATPPO-T algorithm achieves the highest reward and fastest convergence, verifying its efficiency and robustness in dynamic vehicular edge computing networks.

**Figure 7:**
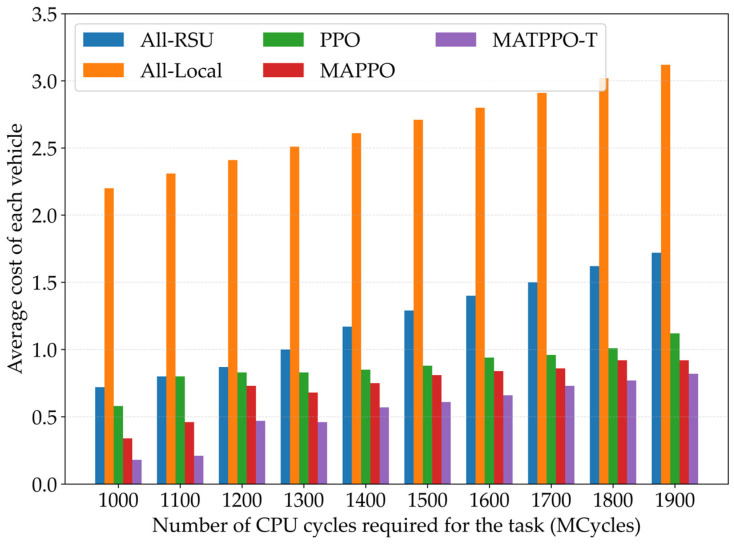
Impact of CPU Cycles Required for Different Tasks on Average Task Offloading Cost.

Figure 7 compares the average cost of each vehicle under different task computations required (MCycles) for five schemes: all-local, all-RSU, PPO, MAPPO, and the MATPPO-T scheme proposed in this paper. According to the figure, the cost in all scenarios gradually increases with the increasing number of CPU cycles required for tasks. Due to the limited CPU resources of local devices, devices need to operate at full load for extended periods, resulting in more significant curve growth in the local execution scenario. In contrast, the curve growth of the proposed MATPPO-T scheme is relatively gentle, and compared with other schemes, the average overhead is lower. This validates the effectiveness of the proposed MATPPO-T scheme.

**Figure 8:**
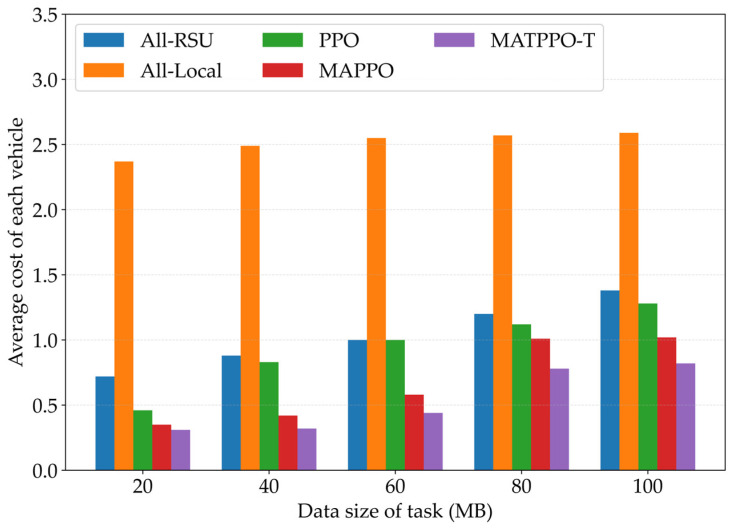
Impact of Different Task Data Sizes on Average Task Offloading Cost.

Figure 8 compares the average cost of each vehicle under different task data sizes for five schemes: all-local, all-RSU, PPO, MAPPO, and the MATPPO-T scheme proposed in this paper. According to the figure, as task data size increases, the all-local execution scheme exhibits the smallest metric fluctuation because the local scheme processes tasks in a fixed order without relying on algorithms for task allocation. However, its performance is the worst; in contrast, the PPO, MAPPO, and MATPPO-T schemes all demonstrate better performance, with the proposed MATPPO-T scheme being the most outstanding, as it can effectively adapt to high-load scenarios and dynamically adjust task allocation strategies to reduce system average overhead. Under high data volume conditions, MATPPO-T can still maintain a relatively low overhead growth rate, indicating its good scalability. The experimental results fully demonstrate the stability and superiority of MATPPO-T under different load conditions.

**Figure 9:**
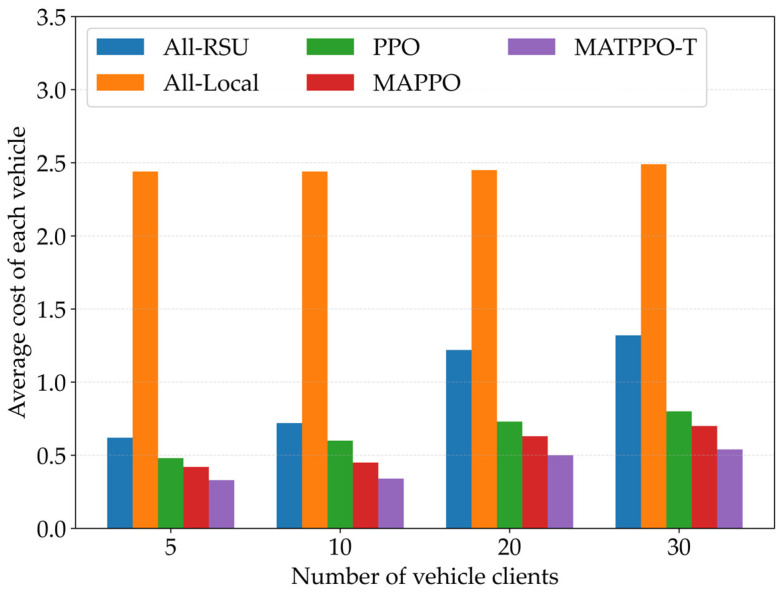
Impact of Different Numbers of Vehicle Clients on Average Task Offloading Cost.

Figure 9 compares the average cost of each vehicle under different numbers of vehicle clients for five schemes: all-local, all-RSU, PPO, MAPPO, and the MATPPO-T scheme proposed in this paper. As the number of vehicles increases, the network topology becomes more complex and resource competition intensifies. The experimental results show that changes in the number of vehicle clients have no impact on all-local computing, as local computing relies solely on vehicle computing resources without occupying computing resources from other servers. The overheads of other schemes show an upward trend, but MATPPO-T exhibits the slowest growth rate and the lowest average overhead. In high-density scenarios, this scheme can still maintain a relatively low cost level, benefiting from its efficient collaborative decision-making mechanism and dynamic load balancing capability.

**Figure 10:**
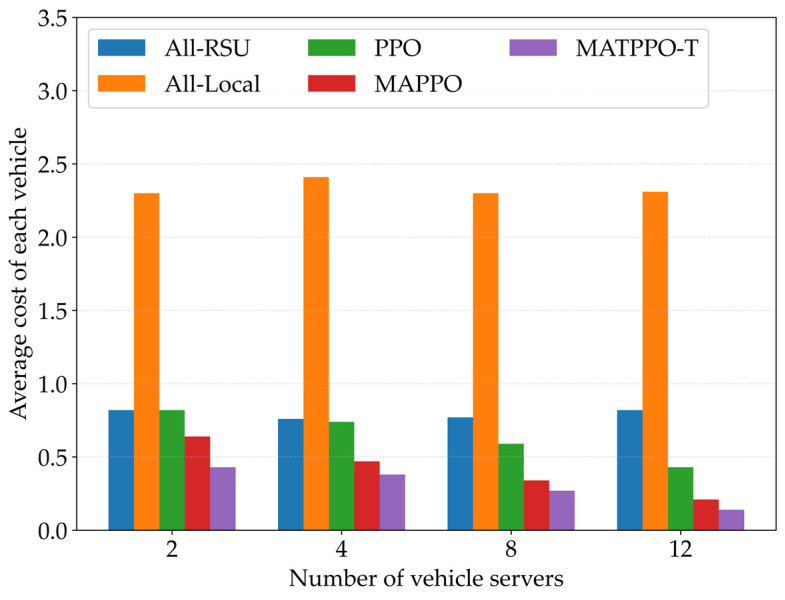
Impact of Different Numbers of Vehicle Servers on Average Task Offloading Cost.

Figure 10 compares the average cost of each vehicle under different numbers of vehicle servers for five schemes: all-local, all-RSU, PPO, MAPPO, and the MATPPO-T scheme proposed in this paper. First, changes in the number of vehicle servers have no impact on all-local computing and all-offloading to RSU; second, the number of vehicle servers is negatively correlated with average task offloading cost. When more vehicle servers are available, the vehicular edge computing network has more computing resources, and offloading computation tasks to nearby service vehicles incurs lower transmission latency. The proposed MATPPO-T scheme consistently maintains lower task offloading costs, which also validates the effectiveness of the proposed scheme.

**Figure 11:**
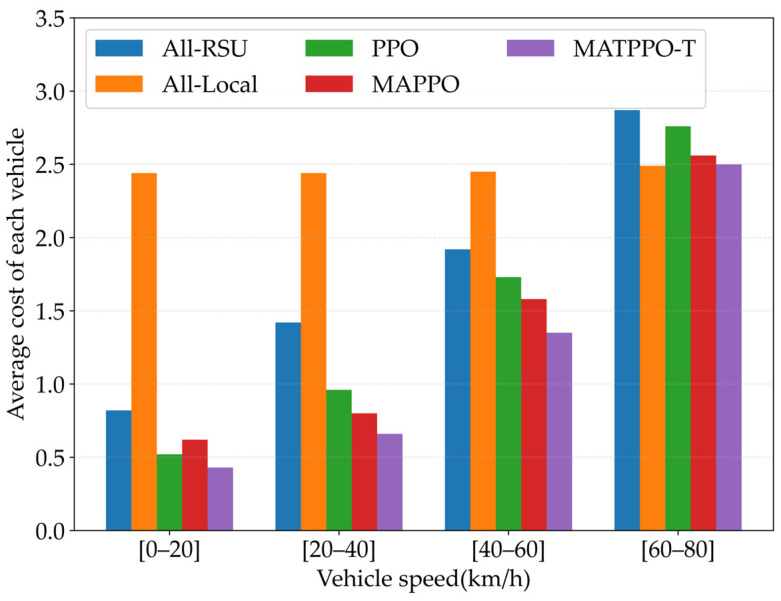
Impact of Different Speeds of Vehicle on Average Task Offloading Cost.

Figure 11 compares the average cost of each vehicle under different vehicle speeds for five schemes: all-local, all-RSU, PPO, MAPPO, and the MATPPO-T scheme proposed in this paper. Vehicle speed has no effect on the all-local strategy since it requires no communication. At low speeds, all-RSU and MAPPO achieve lower costs; at high speeds, costs increase significantly due to vehicles leaving RSU coverage and triggering cross-zone result migration, which increases I2I link transmission latency overhead. MATPPO-T maintains the lowest cost across all speed intervals with the slowest growth rate, thanks to the Transformer-based dual Critic network for capturing global heterogeneous features and suppressing noise, as well as the proactive prediction of vehicle mobility trends and migration path optimization enabled by embedded result migration decisions.

**Figure 12:**
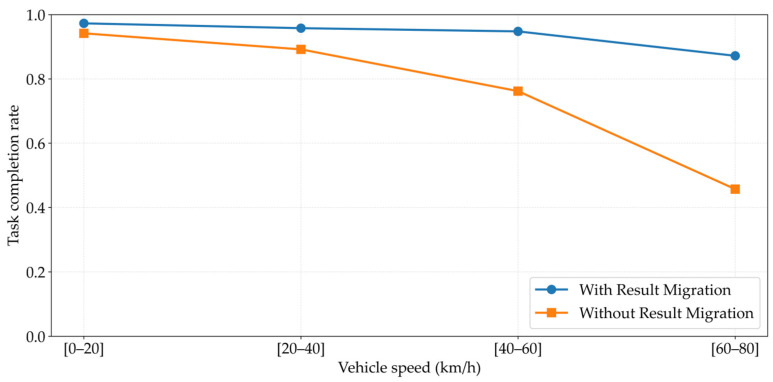
Impact of Result Migration Mechanism on Task Completion Rate under Different Vehicle Speeds.

Figure 12 analyzes the impact of the result migration mechanism on task completion rate across different vehicle speed intervals. Without result migration, the task completion rate drops significantly as speed increases: plummeting to 46% in high-speed scenarios ([60–80] km/h), a 48-percentage-point decrease compared to low-speed scenarios. With result migration enabled, completion rates stabilize at 87–98% across all speed intervals, improving reliability by approximately 41 percentage points in high-speed scenarios. Notably, 8–12% of tasks still fail above [40–60] km/h; this is not a mechanism failure but rather stems from the physical limit where high-speed vehicles move beyond all-RSU coverage ranges—a fundamental difference from failures caused by mechanism absence without migration.

**Figure 13:**
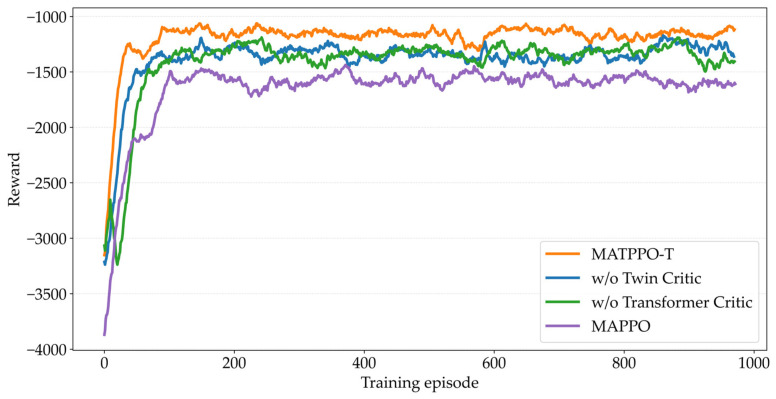
Comparison of ablation studies.

To deeply analyze the impact of key components of the MATPPO-T algorithm on performance in vehicular edge computing offloading, we respectively remove the Transformer-based Critic network structure and twin Critic mechanism from MATPPO-T to verify each module’s contribution to offloading strategy learning. The experimental settings are as follows: (1) full implementation of MATPPO-T algorithm (MATPPO-T); (2) MATPPO-T without twin Critic mechanism (w/o twin Critic), using single Critic network for value estimation; (3) MATPPO-T without Transformer Critic (w/o Transformer Critic), replacing the global state encoder with fully connected network; (4) baseline algorithm MAPPO. The experimental results are shown in Figure 13: the twin Critic mechanism is crucial for convergence stability. After removal, the model converges slower with increased curve fluctuations, indicating that a single Critic struggles to resist noise interference from vehicle mobility and channel fluctuations, easily leading to value overestimation. The Transformer Critic significantly enhances state representation capability. After removal, performance drops noticeably, showing that fully connected networks fail to capture high-order coupling among four heterogeneous features, while Transformer’s attention mechanism effectively captures complex dependencies in dynamic environments. Comparison with MAPPO validates the superposition effect of improvements, with both mechanisms synergistically supporting MATPPO-T’s fast convergence and low offloading cost.

### 6.3. Computational Cost Analysis

In terms of computational complexity, PPO has the lowest complexity as a single agent algorithm, while MAPPO has increased computational complexity due to the introduction of multi-agent collaborative decision making. However, the MATPPO-T proposed in this paper has slightly higher single step training complexity due to the use of a dual Transformer Critic structure and centralized training framework. However, thanks to the efficient modeling of global heterogeneous states such as queue load, communication links, task attributes, and computing resources by Transformer, as well as the significant suppression of value overestimation problems by the dual Critic minimum mechanism, MATPPO-T can converge faster and stably, effectively reducing total training time and resource consumption. Table 5 shows the total running time of each algorithm under different numbers of vehicle clients. It can be seen that, compared with benchmark algorithms such as PPO and MAPPO, the method proposed in this paper only introduces limited additional overhead, but the above experimental results show significant performance improvements in indicators such as average task offloading cost and convergence speed. Therefore, the trade-off between performance and computational overhead of the algorithm is reasonable, which can be applied to the actual vehicle edge computing network.

## 7. Conclusions

This paper conducts in-depth research on the computation offloading optimization problem in multi-vehicle vehicular edge computing network (VECN) scenarios. To address the core characteristics of vehicular tasks—namely, latency-sensitive and computation-intensive—as well as the result return challenges caused by limited system resources and vehicle mobility, we construct a complete system model encompassing multiple service nodes, multiple communication links, and multiple computing modes. We propose the MATPPO-T algorithm based on multi-agent deep reinforcement learning, forming a comprehensive task offloading solution that balances latency and energy consumption optimization. Through systematic theoretical analysis and extensive simulation experiments, the effectiveness and superiority of the proposed scheme are comprehensively validated. Experiments demonstrate that MATPPO-T converges faster, achieves lower offloading costs, and exhibits better stability and scalability than benchmark schemes such as PPO and MAPPO under different task characteristics and network scales. This method is validated under simplified road network and ideal communication assumptions, leaving room for improvement in adapting to complex road topologies and non-ideal channels. Future work may incorporate more realistic vehicular communication environments and dynamic road network characteristics to further enhance the algorithm’s generalization capability and robustness, expanding its practical application in large-scale vehicular networks.

## Figures and Tables

**Table 1:** Summary and comparison of related works.

Reference	Contribution	Limitations	Our Work
[12]	Game theory combined with deep reinforcement learning for autonomous offloading decision.	The single-agent perspective fails to characterize the policy coupling when multiple vehicles offload simultaneously.	Adopt multi-agent reinforcement learning to explicitly model the coupling relationships in multi-vehicle collaborative decision making.
[13]	Meta-learning improves model adaptability.	The risk of cross-zone communication failure caused by vehicle mobility is overlooked.	We introduce result migration to proactively handle delivery failures when vehicles leave RSU coverage.
[14]	Fuzzy reasoning dynamically adapts optimization goals.	Single Critic network suffers from large oscillations under high dynamics.	Design a twin Critic network architecture that takes the minimum value to suppress mobility-induced noise and reduce policy gradient variance.
[16]	MAPPO algorithm for resource allocation and task offloading.	Single Critic network fully connected network, lacking mechanisms to prevent overestimation.	Dual Critic network architecture suppresses overestimation issues.
[18]	Resource allocation is optimized via an enhanced genetic algorithm.	Static models struggle to adapt to dynamic environments.	Online dynamic decision making is achieved via multi-agent reinforcement learning to adapt to dynamic environments.
[19]	The offloading position and strategy are optimized via quantum-behaved particle swarm optimization.	Heuristic algorithms suffer from high computational complexity, failing to meet real-time requirements; moreover, they lack modeling of result return links.	End-to-end reinforcement learning is adopted to enable rapid decision making while explicitly modeling the result return link.
[20]	A volunteer-assisted vehicular edge computing framework is proposed, where a Stackelberg game and genetic algorithm are employed to jointly optimize offloading decisions and resource allocation.	Game-theoretic methods rely on full information assumptions that are hard to meet in realistic scenarios, and they neglect the influence of vehicular mobility on computation result delivery.	Works under partial observability assumptions, eliminating the need for full information; incorporates result migration decisions to cope with vehicular mobility.
[21]	Ant colony optimization-based task offloading and load balancing strategy for fog computing with response time as the optimization objective.	Heuristic algorithms suffer from slow convergence, making them ill-suited for rapidly changing vehicular environments; furthermore, the overhead of returning computation results is overlooked.	MATPPO-T algorithm is employed for fast convergence, enabling joint optimization of result migration decisions.
[22]	Vehicular tasks are decomposed into subtasks, and system average latency is minimized based on ant colony optimization.	Decomposing tasks incurs extra coordination costs; moreover, static optimization approaches fail to adapt to time-varying network topologies.	Binary offloading is used to lower coordination overhead, with online learning enabling adaptation to dynamic topology changes.
[25]	MAPPO algorithm implementation for RIS-assisted vehicular edge computing.	The Critic network adopts a fully connected structure, making it difficult to capture heterogeneous feature correlations.	Transformer encodes global states, twin Critics suppress noise, better suited for high-speed mobile vehicular scenarios.

**Table 2:** Principal notations.

Parameters	Value
$D_{i}$	Size of task data
$C_{i}$	Number of CPU cycles required to complete the task
$T_{max}$	Maximum tolerable execution time for the task
$R_{n,rsu}$	Data transmission rates between vehicle client and MEC server
$R_{n,m}$	Data transmission rates between vehicle client and vehicle server
$R_{j,k}$	Data transmission rates between MEC server and MEC server
B	Channel bandwidth
$N_{0}$	Gaussian noise power within the channel
$P_{n}$	Transmit power of vehicle client
$P_{m}$	Transmit power of vehicle server
$P_{rsu}$	Transmit power of MEC server
$h_{k}$	Channel gain
$\eta_{task}$	Task data processing coefficient
$f_{n}$	Computing capabilities of vehicle client local terminal
$f_{m}$	Computing capabilities of vehicle server
$f_{rsu}$	Computing capabilities of MEC server
$k_{n},\;k_{m}$	Effective switching capacitance of vehicle client and vehicle server
$o_{n}$	Unit resource cost
μ	Actual number of tasks arriving at the vehicle server per unit time
$p_{r}$	Computing power of MEC server

**Table 3:** Simulation parameters.

Parameters	Value
Number of vehicle clients	[5, 30]
Number of vehicle servers	[2, 12]
Number of RSUs	6
Vehicle speed (km/h)	[10, 80]
MEC server device power (W)	25
Vehicle client device power (W)	4
Vehicle server device power (W)	4
Task data size constraint (MB)	[0, 100]
Task latency constraint (s)	[0, 1]
CPU cycles required per bit (cycles/bit)	[1000, 1900]
Vehicle computing capability (GHz)	2
MEC server computing capability (GHz)	18

**Table 4:** Hyperparameter settings for MATPPO-T.

Parameters	Value
Actor network learning rate	0.001
Critic network learning rate	0.001
Clip threshold	0.1
Entropy regularization coefficient	0.001
Batch size	64
Number of training episodes	50,000
Number of steps	60
Number of hidden layers	2
Hidden layer neuron size	128

**Table 5:** Comparison of running time of different algorithms.

Numbers of Vehicle Clients	PPO	MAPPO	MATPPO-T
5	0.48	0.67	0.91
10	2.16	2.66	3.02
20	2.52	3.19	3.68
30	3.27	4.01	4.66

## Data Availability

Data are contained within the article.
